# Supplementary material for: iModMix: integrative module analysis for multi-omics data
Source: Bioinformatics. 2026 Jan 19;42(2):btag030. doi: 10.1093/bioinformatics/btag030 (PMC12925246; doi:10.1093/bioinformatics/btag030)
Supplement: btag030_Supplementary_Data [file btag030_supplementary_data.zip › Supplementary_TechNote_IModMix.pdf]

Case Study S1. Clear cell renal cell carcinoma (ccRCC, RC20) Dataset with Identified Metabolites

We used 24 normal and 52 tumor clear cell renal cell carcinoma (ccRCC) samples (Golkaram et al. 2022; Tang et al. 2023; Benedetti et al. 2023) as a case study. It contained 23001 genes from RNA-seq and 904 identified metabolites from untargeted metabolomics. Applying iModMix identified 751 gene modules and 34 metabolite modules. Differential expression analysis of the modules through t-test confirmed changes in metabolite abundance between groups recapitulated those from prior work (Benedetti et al. 2023) highlighting reduced levels of gamma-glutamyltyrosine (module ME#BA3241, P-value: 0.0003), creatinine/C00791 (module ME#C06162 P-value: 4.2681E-15), xanthosine/C01762 (module ME#66628D P-value: 0.0015), docosahexaenoate (DHA; 22:6n3)/C06429 (module ME#904A67 P-value: 4.6906E-12), and 1-methyladenosine/C02494 (module ME#E1C62F P-value: 8.1847E-14) in tumors compared to normal tissue. Conversely, metabolites with increased abundance in tumors compared to normal tissues included proline/C00148, glutamine/C00064 (module ME#6C856F, P-value: 0.006), maltose/C00208 and 1-methylnicotinamide/C02918 (module ME#904A67 P-value: 4.6906E-12) (Benedetti et al. 2023). The top five most correlated metabolomic and transcriptomic module pairs included pathways such as Inflammatory Response (P-value: 0.0001), Protein Polyubiquitination (P-value: 0.0001), tRNA Aminoacylation for Mitochondrial Protein Translation (P-value: 0.0084), and Sphingosine-1-Phosphate Receptor Signaling Pathway (P-value: 0.0068). The inflammatory response plays a crucial role in the development and prognosis of ccRCC. Research indicates that inflammation is involved at all stages of the disease, influencing tumor progression and treatment responses (Zhong et al. 2023).

Case Study S2. Lung Adenocarcinoma Dataset with Unidentified Metabolites

Matched proteomics and metabolomics dataset was generated using two mouse models for lung adenocarcinoma (LUAD) (10 wild type, 10 knockout), with 7353 metabolomics features (identified and unidentified) and 7928 protein groups. Applying iModMix generated 412 gene modules, and 287 metabolite modules. Strong correlations were observed between these modules, with correlations as high as 0.93. The correlations observed between variables in the 2 and 3 correlated pairs (Table S1) are notably high, with numerous correlations between proteins and both identified and unidentified metabolites exceeding 0.9 (Fig. S1A, Fig. S1B). These strong correlations offer valuable insights into the unidentified metabolites, providing crucial information about their potential relationships and helping to elucidate their behavior. Pathway analysis of these 16 genes from and the identified metabolite using MetaboAnalyst (Pang et al. 2024) revealed significant enrichment in Proteoglycans in cancer (P-value: 7.9492E-4) and Renin-angiotensin system (P-value: 7.9861E-4). Proteoglycans are critical in LUAD, influencing tumor progression, metastasis, and microenvironment. Their dysregulation impacts essential cellular processes such as epithelial-to-mesenchymal transition and cancer stemness, which are key to cancer cell plasticity and aggressiveness (Karagiorgou et al. 2022). Besides, focal adhesions play a significant role in LUAD progression by mediating cell adhesion, migration, and signaling pathways essential for tumorigenesis. Studies highlight the involvement of focal adhesion kinase (FAK) in LUAD, suggesting potential therapeutic targets and biomarkers for this cancer type (Zhou et al. 2018).

Supplementary Table 1. Top 3 modules Case Study 2: Results from iModMix analysis of Lung Adenocarcinoma (LUAD) dataset with unidentified metabolites highlighting the top correlated metabolomic-proteomic modules.

Rank	Metabolite Module	# of var	Metabolites	Protein Module	# of var	Proteins	Corr
1	#BF862B	18	0 identified	#CF8BAF	11	Pdgfra, Klhl15, Stx2, Rtkn2, Ager, Gypc, Erbin, Rap2a, Abca8b, Lims2, Cav2	0.9293
2	#FFB214	21	7 identified:<br>C00166: Phenylpyruvic acid,<br>C00954: Indoleacetic acid,<br>C05582: Homovanillic,<br>C03672: Hydroxyphenyllactic acid,<br>C01179: 4Hydroxyphenylpyruvic,<br>C00526: Deoxyuridine,<br>C00178: Thymine	#FFDD25	16	Myl6, Tmod1, Vsnl1, Tgfb1i1, Ppp1r14a, Aldh1a1, Ilk, Myl12b, Dlc1, Plscr4, Macf1, Pcdh18, Limch1, Lims1, Specc1l, Vcl, Rasip1	0.9293
3	#5E985E	26	1 identified:<br>C06426: gamma-linolenic acid	#FFDA24	16	Hsd11b1, Cavin2, Ehd2, Myh10, Clic5, Msn, Myo1c, Epb41l2, Pakap, Sptan1, Akap12, Cyp2b10, Cav1, Cav3, Sptbn1, Ace, Ace3, Cavin1	0.9278

Supplementary Figure 1. Top modules Case Study 2: Results from iModMix analysis of Lung Adenocarcinoma (LUAD) dataset using metabolomics with unidentified metabolites. Results highlight the top correlated metabolomic-proteomic modules with unidentified metabolites.

A) Most correlated modules: Top 2

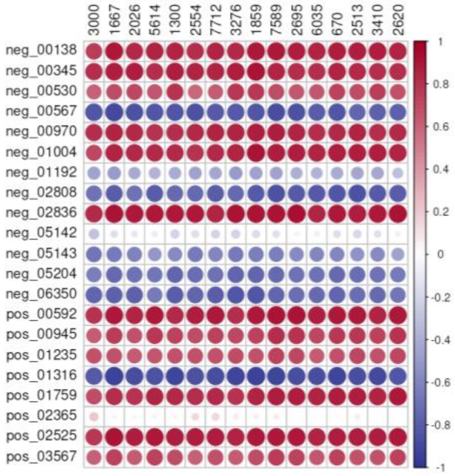

Modules correlation: Data 1 and Data 2

Show 10 entries Search:

	Data1	Data2	Correlation
203	pos_02119	699	0.9414
204	pos_02244	699	0.9414
369	neg_00999	1023	0.9338
126	pos_02244	4567	0.9278
334	pos_02244	1668	0.9248
48	pos_02244	3331	0.9233
125	pos_02119	4567	0.9233
308	pos_02244	1103	0.9218
411	pos_02119	598	0.9218
412	pos_02244	598	0.9218

Showing 1 to 10 of 416 entries Previous 1 2 3 4 5 … 42 Next

Features from Data 1

	Feature_ID	Metabolite
4	neg_00567	Thymine
10	neg_05142	Phenylpyruvate
13	neg_06350	Indole-3-acetate
7	neg_01192	Homovanillate|3-(4-Hydroxyphenyl)lactate
8	neg_02808	Deoxyuridine
11	neg_05143	3-(4-Hydroxyphenyl)pyruvate

Features from Data 2

	Feature_ID	Symbol
1	1300	Aldh1a1
2	1667	Tmod1
3	1859	Plscr4
4	2026	Vsnl1
5	2513	Specc1l
6	2554	Myl12b
7	2620	Rasip1
8	2695	Limch1
9	3000	Myl6
10	3276	Tgfb1i1

	Feature_ID	Symbol
11	3410	Vcl
12	5614	Ppp1r14a
13	6035	Lims1
14	670	Ilk
15	7589	Macf1 Pcdh18
16	7712	Dlc1

B) Most correlated modules: Top 3

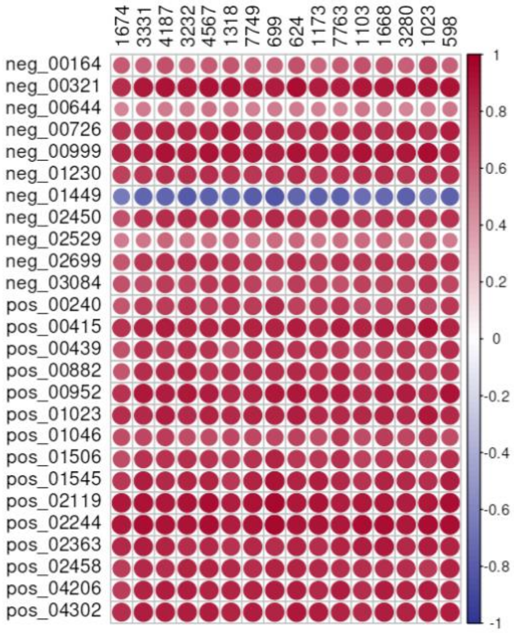

Modules correlation: Data 1 and Data 2

Show 10 entries Search:

	Data1	Data2	Correlation
38	pos_01316	1667	-0.9338
185	pos_01316	1859	-0.9323
219	neg_[illegible]836	2695	0.9218
41	pos_02525	1667	0.9158
164	pos_01316	3276	-0.9158
30	neg_02836	1667	0.9143
198	neg_02836	7589	0.9128
101	pos_01316	1300	-0.9113
203	pos_00592	7589	0.9113
269	pos_01316	670	-0.9113

Showing 1 to 10 of 336 entries Previous 1 2 3 4 5 … 34 Next

Features from Data 2

	Feature_ID	Symbol
1	1023	Ace Ace3
2	1103	Cyp2b10
3	1173	Sptan1
4	1318	Msn
5	1668	Cav1 Cav3
6	1674	Hsd11b1
7	3232	Myh10
8	3280	Sptbn1
9	3331	Cavin2
10	4187	Ehd2

	Feature_ID	Symbol
11	4567	Clic5
12	598	Cavin1
13	624	Pakap
14	699	Epb41l2
15	7749	Myo1c
16	7763	Akap12

Features from Data 1

	Feature_ID	Metabolite
17	pos_01023	Gamma-linolenic acid

Supplementary Figure 2. Running time of iModMix. The running time depends on the size and complexity of the dataset. Here we show 3 datasets demonstrating an iModMix run can be completed in around 10 minutes: Data 1: 7323 variables and 20 samples; Data 2: 7928 and 20 samples; Data 3: 23000 variables and 77 samples, can be completed in around 10 minutes

Data 1: 7323 variables

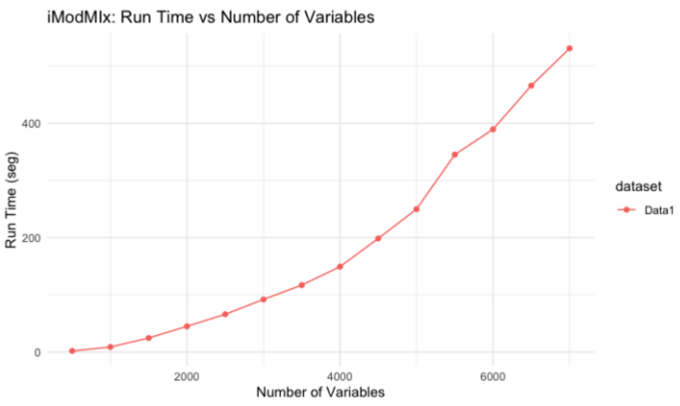

Data 2: 7928 variables

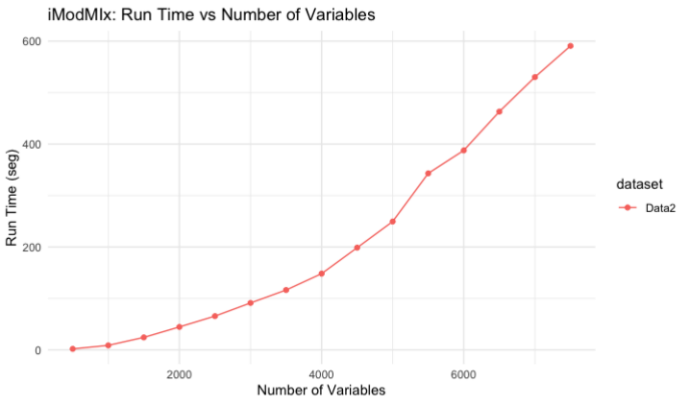

Data 3: 23000 variables

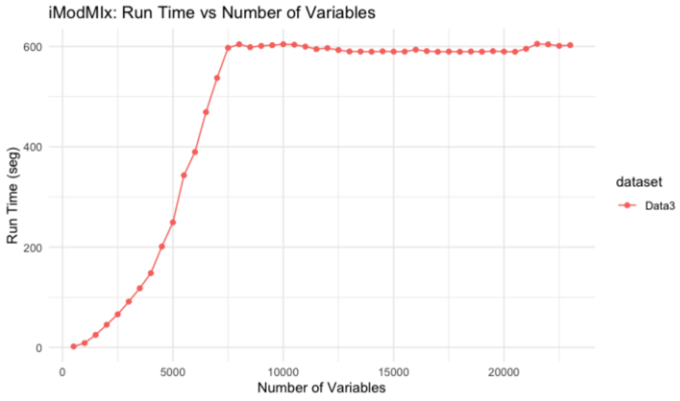

Data 1, Data 2 and Data 3

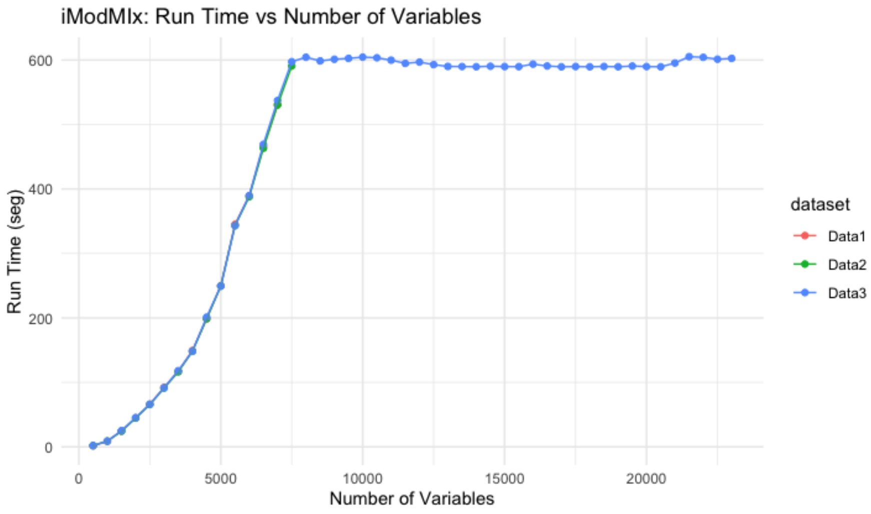

Supplementary Figure 3. Benchmaking results. Benchmarking using clear cell renal cell carcinoma pubic available data (ccRCC, RC20) and lung adenocarcinoma (LUAD) data results demonstrates the performance of lambda vs time for number of genes. Stability was achieved in both datasets with an alpha value of 0.25

A) Metabolomic abundance ccRCC (RC20 dataset)

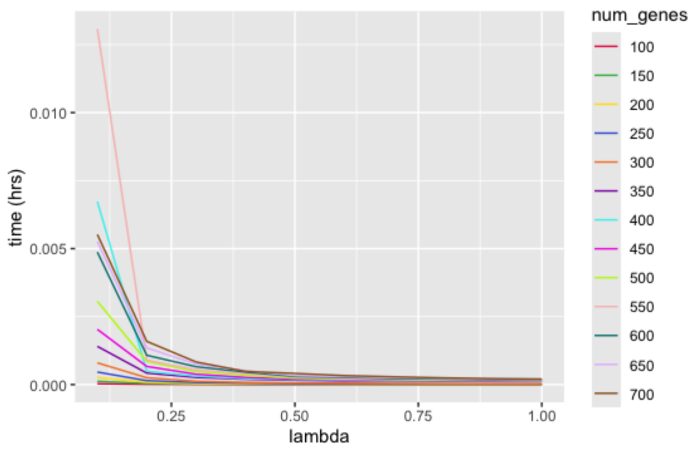

B) Gene expression lung adenocarcinoma (LUAD) data

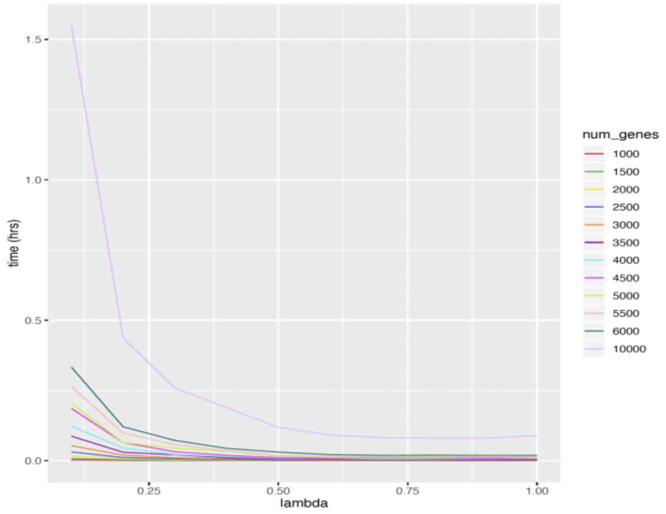

C) Gene expression lung adenocarcinoma (LUAD) data

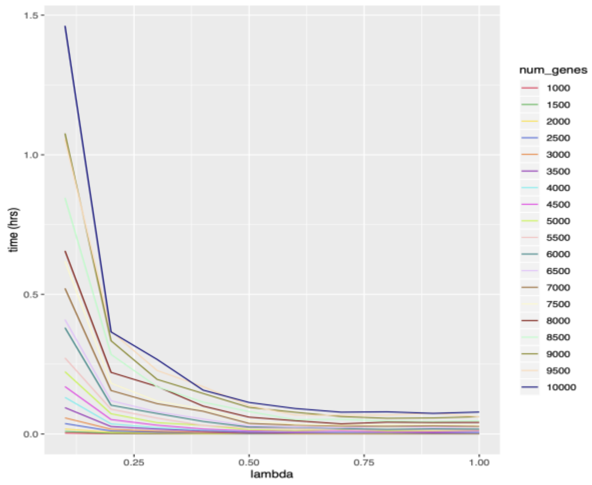

Supplementary Table 2. WGCNA vs iModMix: Number of modules generated from lung adenocarcinoma (LUAD) data. For metabolomics data, WGCNA generated 9 modules (Mean size = 817), whereas iModMix produced 287 modules (Mean size= 23). In the case of proteomics data, WGCNA generated 22 modules (Mean size= 360), while iModMix identified 412 modules (Mean size= 17). iModMix resulted in more modules with a smaller average number of features per module.

Dataset	# of Features	WGCNA		iModMix	
		# Modules	Average of features per module	# Modules	Average of features per module
Metabolites	7,353	9	817	287	23
Proteins	7,928	22	360	412	17

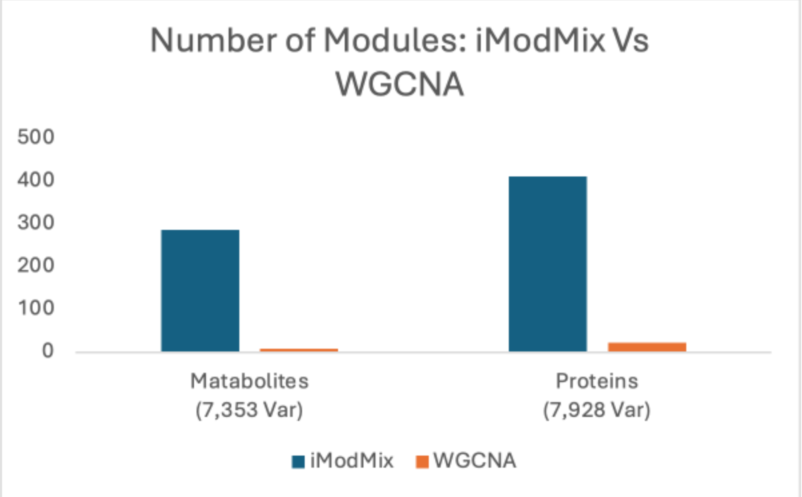

Supplementary Figure 4. WGCNA vs iModMix: Modules distribution in lung adenocarcinoma (LUAD) data shows iModMix resulted in smaller and more uniformly sized modules. For metabolomics data, WGCNA generated 9 modules (Mean size = 817, SD = 2206), whereas iModMix produced 287 modules (Mean size= 23, SD = 11.2). In the case of proteomics data, WGCNA generated 22 modules (Mean size= 360, SD = 559), while iModMix identified 412 modules (Mean size= 17, SD = 5.75).

Metabolomics LUAD data

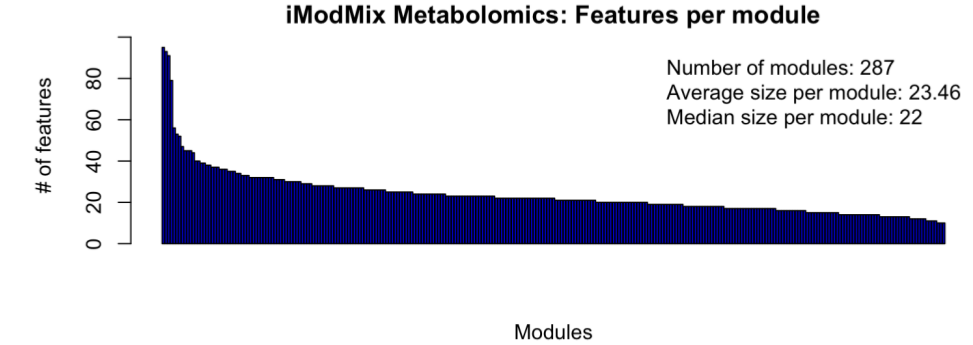

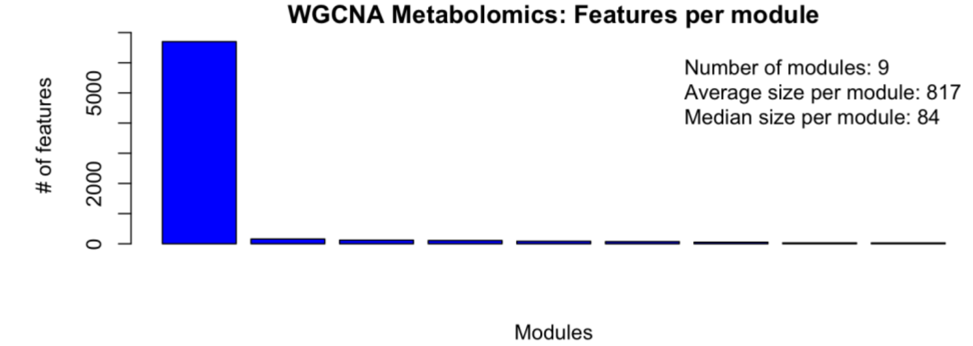

Proteomics LUAD Data

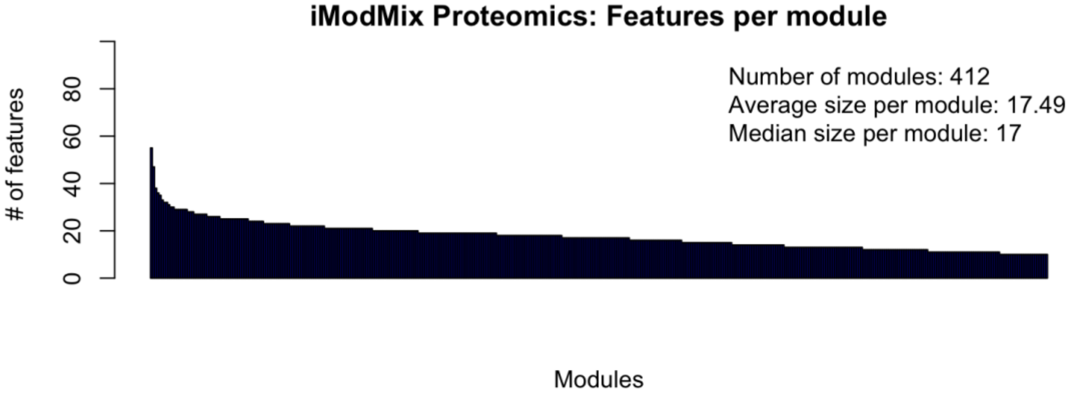

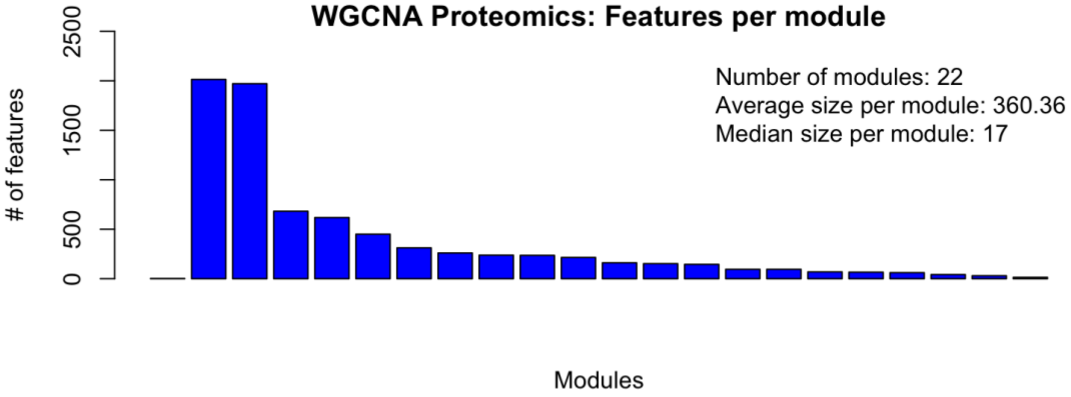

Supplementary Figure 5. WGCNA vs iModMix: Modules distribution in lung adenocarcinoma (LUAD) data. The standard deviation for WGCNA modules was very high, indicating a wide range of module sizes, whereas iModMix modules were more uniform in size (Fig. S3).

Metabolomics data

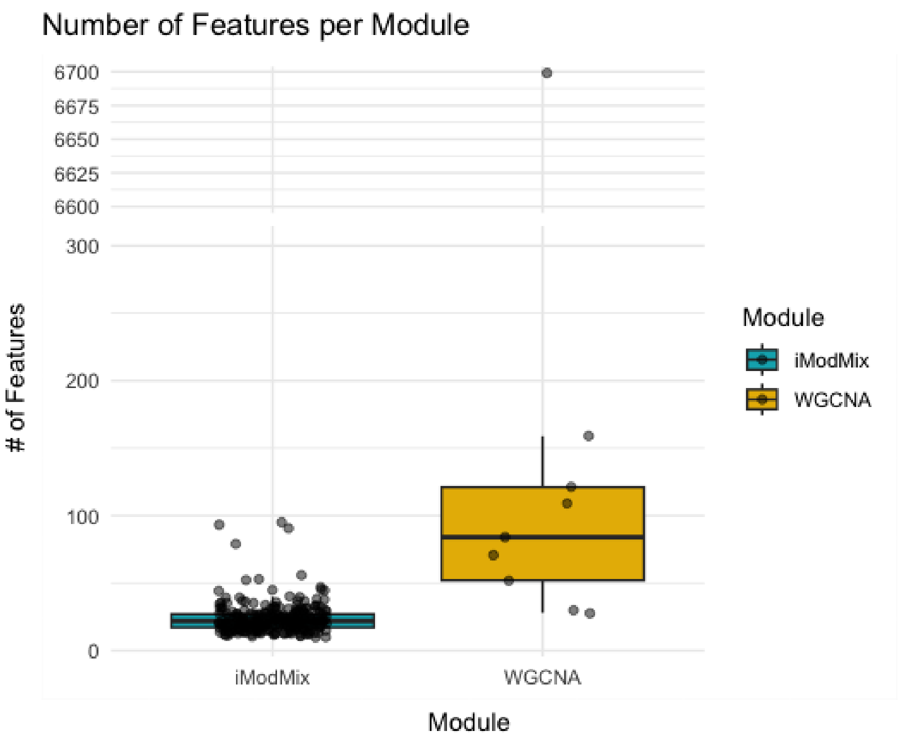

Proteomics Data

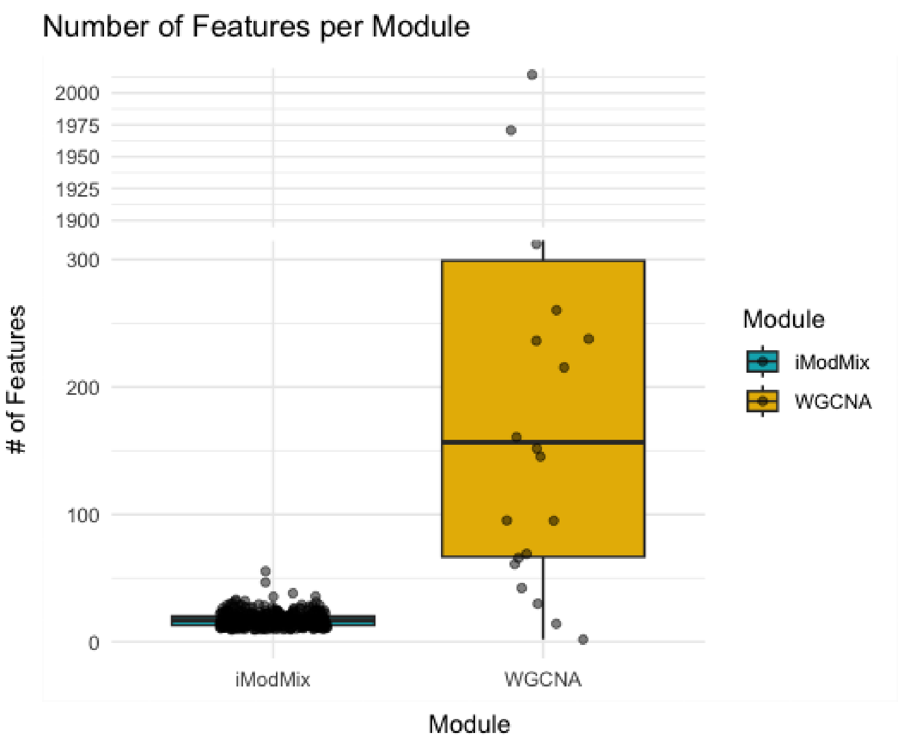

Supplementary Figure 6. WGCNA vs iModMix: Enrichment Analysis by Enrichr for proteins modules using the lung adenocarcinoma (LUAD) data. iModMix resulted in smaller more uniformly sized modules, identifying 181 pathways (provided as a supplemental spreadsheet, Table S3) from protein modules compared to the 13 pathways identified by WGCNA. Notably, all 13 pathways identified by WGCNA were also detected by iModMix.

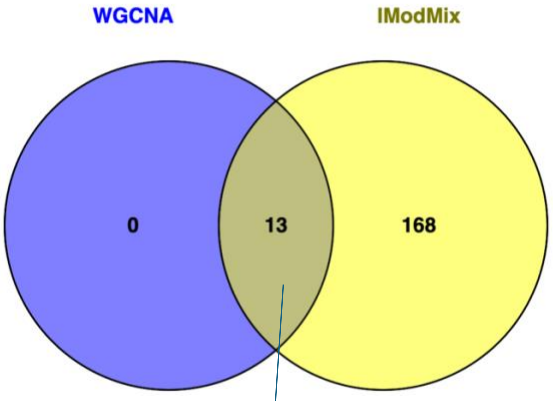

**Commons**
Ubiquitin mediated proteolysis
Platelet activation
Complement and coagulation cascades
Epstein-Barr virus infection
Valine, leucine and isoleucine degradation
Spliceosome
ECM-receptor interaction
Cardiac muscle contraction
Protein export
Oxidative phosphorylation
Metabolism of xenobiotics by cytochrome P450
IL-17 signaling pathway
Ribosome

Supplementary Figure 7. Integration of omics data using WGCNA Vs iModMix. Correlation plot from the integration process between metabolite and protein modules using the lung adenocarcinoma (LUAD) data. The top correlation achieved using iModMix was 0.93, whereas WGCNA reached a top correlation of 0.88.

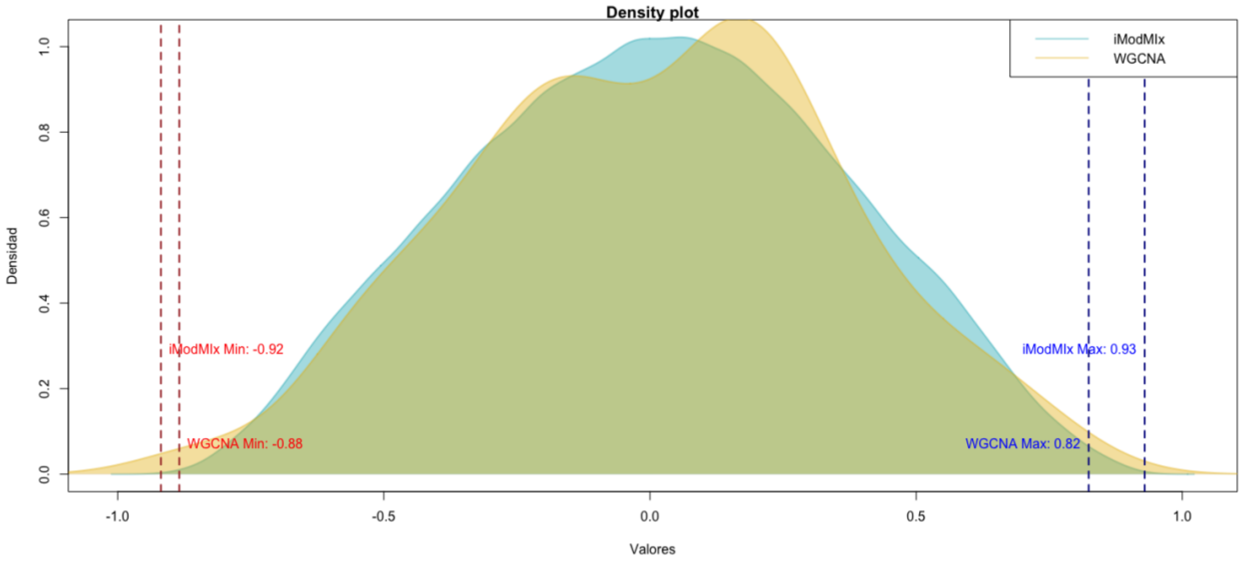

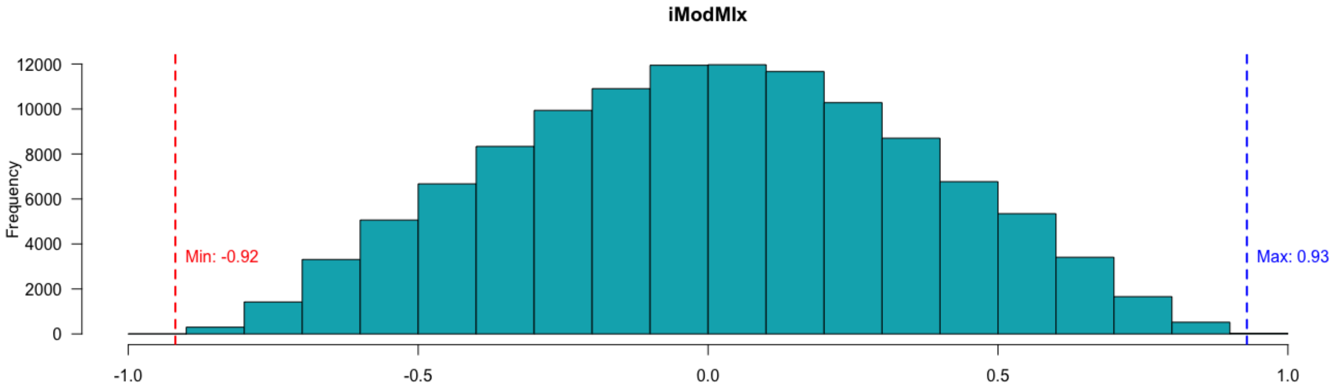

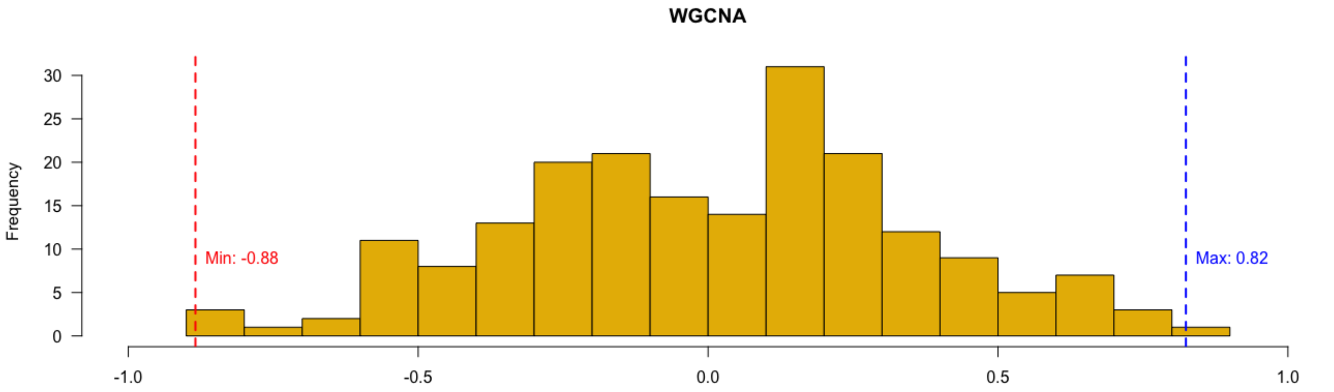

Top 5 correlations from integration

Data1	Data2	Correlation
D1#red	D2#magenta	-0.8842
D1#black	D2#magenta	-0.8496
D1#turquoise	D2#turquoise	0.8241
D1#brown	D2#magenta	-0.8045
D1#brown	D2#green	0.7594

Data1	Data2	Correlation
D1#BF862B	D2#CF8BAF	0.9293
D1#FFB214	D2#FFDD25	0.9293
D1#5E985E	D2#FFDA24	0.9278
D1#B83343	D2#DD1D22	0.9248
D1#EF7718	D2#DD1D22	0.9248

Supplementary Table 4. A comparative overview of the underlying principles and analytical frameworks of iModMix, PIUMet, iClusterPlus, and WGCNA.

Feature / Method	iModMix	WGCNA	PIUMet	iClusterPlus
**Integration of multi-omics data**	Yes	No	Yes	Yes
**Supports Horizontal integration of multi-omics data**	Yes	Yes	Yes	No
**Processes any number of omics datasets simultaneously**	Yes	No	No	No
**Handling of unidentified metabolites**	Yes	No	Yes	No
**Employes network-based approach**	Yes	Yes	Yes	No
**Is not constrained by prior knowledge (e.g., seeds)**	Yes	Yes	No	Yes
**Identifies co-expression modules**	Yes	Yes	No	No
**Useful for feature selection**	Yes	Yes	Yes	Yes
**Provided as an R package**	Yes	Yes	No	Yes
**Allow to unveil novel connections**	Yes	Yes	Yes	Yes
**User-friendly (Shiny app)**	Yes	Yes	No	No
**Scores (Total Yes)**	11	8	6	5
